# Co-Variation of Tonality in the Music and Speech of Different Cultures

**DOI:** 10.1371/journal.pone.0020160

**Published:** 2011-05-27

**Authors:** Shui' er Han, Janani Sundararajan, Daniel Liu Bowling, Jessica Lake, Dale Purves

**Affiliations:** 1 Neuroscience and Behavioral Disorders Program, A*STAR Neuroscience Research Partnership, Duke-NUS Graduate Medical School, Singapore, Singapore,; 2 Department of Neurobiology, Duke University Medical Center, Durham, North Carolina, United States of America,; 3 Center for Cognitive Neuroscience, Levine Science Research Center, Duke University, Durham, North Carolina, United States of America,; 4 Department of Psychology and Neuroscience, Duke University, Durham, North Carolina, United States of America; Duke University, United States of America

## Abstract

Whereas the use of discrete pitch intervals is characteristic of most musical traditions, the size of the intervals and the way in which they are used is culturally specific. Here we examine the hypothesis that these differences arise because of a link between the tonal characteristics of a culture's music and its speech. We tested this idea by comparing pitch intervals in the traditional music of three tone language cultures (Chinese, Thai and Vietnamese) and three non-tone language cultures (American, French and German) with pitch intervals between voiced speech segments. Changes in pitch direction occur more frequently and pitch intervals are larger in the music of tone compared to non-tone language cultures. More frequent changes in pitch direction and larger pitch intervals are also apparent in the speech of tone compared to non-tone language cultures. These observations suggest that the different tonal preferences apparent in music across cultures are closely related to the differences in the tonal characteristics of voiced speech.

## Introduction

Tonal differences between traditional Eastern and Western music are readily heard. Explanations often refer to the use of different scales [1]–[3], but this begs the question of why different sets of pitch intervals are preferred in the first place. The alternative we examine here is that the tonal characteristics of a culture's music are related to the prosodic characteristics of its speech. There are several reasons for entertaining this idea. First, both speech prosody and the melodic contour are used to convey emotion [4]. Second, speech is the principal source of pitch and pitch relationships in the human auditory environment [5]. Third, several aspects of musical tonality including interval preference, scale preference, and the affective impact of major and minor modes are closely tied to voiced speech [5]–[7]. Finally, rhythm and pitch patterns in Western instrumental music and speech are similar [8]–[9].

The use of pitch in speech varies greatly among languages. The most obvious example is the broad division of languages into “tone” and “non-tone” groups [10]. In tone languages, the lexical meaning of each syllable is conveyed by the use of pitch contours, relative pitch levels, or both. For example, Standard Mandarin uses four tones, referred to as “high”, “rising”, “falling then rising” and “falling”; the syllable ‘ma’ uttered as a high tone means ‘mother’, as a rising tone ‘hemp’, with a falling then rising tone ‘horse’, and as a falling tone ‘scold’. Other tone languages, such as Thai and Vietnamese, are similar by definition, but vary in detail, using five and six tones respectively to convey the lexical meaning of syllables [11]–[13]. In contrast, pitch contours and relative levels are not typically used in non-tone languages (e.g., English, French and German) to convey lexical meaning (although stress, which can influence pitch, determines different meaning in some instances, e.g., CONtent or conTENT [12]–[14]). Imbuing each syllable with a different pitch contour gives tone language speech a “sing-song” quality. Accordingly, Standard Mandarin speech has more frequent changes in pitch direction and greater rates of pitch change than American English speech [15].

Based on these differences, we asked how, if at all, differences in the use of pitch in the traditional music of tone and non-tone language speaking cultures compares with the use of pitch in speech. To address this question, we compiled databases of speech and music from several tone and non-tone language cultures. The analysis focused on two aspects of pitch dynamics: the frequency of changes in pitch direction (slope reversals), and the size of the pitch intervals used. These aspects were chosen because they differentiate tone and non-tone language speech and play a central role in the structure of musical melodies [15], [16].

## Results

### Slope Reversals

**Figure 1:**
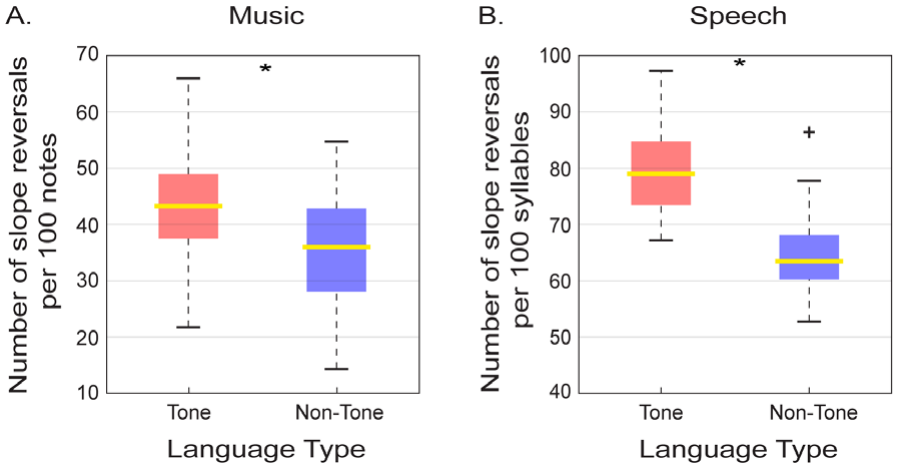
Slope reversals in the music and speech of tone and non-tone language speaking cultures. (A) Box plot showing the distribution of the number of melodic slope reversals per melody in the tone (red) and non-tone (blue) language music databases (*n_1_* = *n_2_* = 90). Horizontal yellow lines indicate medians; colored boxes specify inter-quartile ranges and dashed lines the ranges without outliers. (B) Box plot of the number of prosodic slope reversals per speaker in the tone and non-tone language speech databases (*n_1_* = *n_2_* = 40). The cross indicates an outlier (defined as greater/lesser than 1.5x the inter-quartile range). (*  =  *P*<0.001; all comparisons were made using a Mann Whitney *U*-test, α = 0.05, two-tailed).

Figure 1 shows the number of melodic slope reversals in the music of tone and non-tone language cultures compared to the number of prosodic slope reversals in speech. The median number of melodic slope reversals per 100 notes is greater in the music of tone compared to non-tone language speaking cultures (43.3 vs. 36.0 respectively, *U* = 9843.5, *P*<0.001; Figure 1A). The median number of prosodic slope reversals per 100 syllables is also greater in the speech of tone compared to non-tone language speaking cultures (79 vs. 63.5 respectively, *U* = 2317, *P*<0.001; Figure 1B). (See Figure S5 for breakdown by individual cultures).

### Interval Size

**Figure 2:**
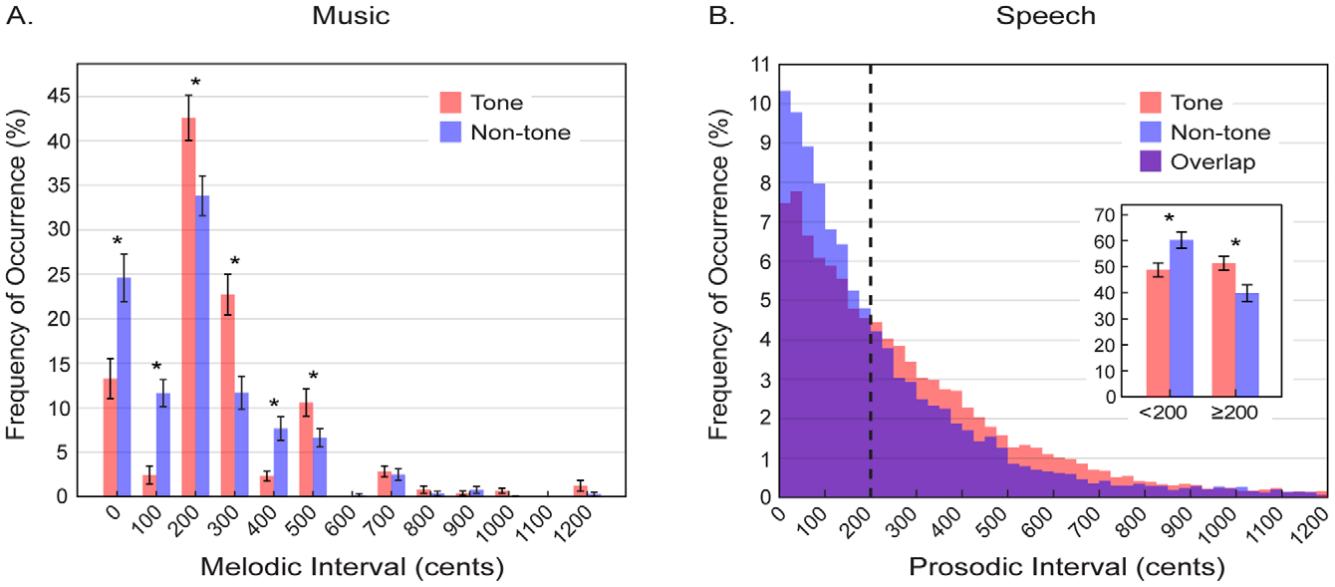
Interval size in the music and speech of tone and non-tone language cultures. (A) The distributions of absolute melodic interval sizes per melody in the tone (red) and non-tone (blue) language music databases (*n_1_* = *n_2_* = 90). (B) The distributions of absolute prosodic interval sizes per speaker in the tone (red) and non-tone (blue) language speech databases. Inset shows the percentages of small (<200 cents) vs. large (≥200 cents) prosodic intervals for tone (red) and non-tone (blue) speech (the vertical dashed line in the main figure separates these groups). Error bars indicate +/−2x standard errors to indicate 95% confidence intervals. (*  =  *P*<0.001; all comparisons are based on a two tailed independent samples *t*-test, α-level adjusted using the Bonferroni method).

Figure 2 shows the size of melodic intervals in the music of tone and non-tone language cultures compared to the size of prosodic intervals in speech. In accord with previous studies [17], [18], the majority of melodic intervals were relatively small (0–500 cents); melodic intervals larger than a perfect fourth (500 cents) were infrequent, presumably because they are harder to sing [19] (Figure 2A). This overall tendency notwithstanding, the average distribution of melodic intervals in the music of tone and non-tone language cultures is different (Table 1). In tone language cultures, intervals smaller than a major second (200 cents) occur less often (15.8% vs. 36.2%; *t* = -11.4, *P*<0.001), whereas intervals equal to or larger than a major second occur more often (84.2% vs. 63.8%; *t* = 11.4, *P*<0.001). The only exception in this overall pattern is major thirds (400 cents), which are more frequent in the music of non-tone language cultures (7.7% vs. 2.3%). Figure 2B shows the average distribution of prosodic interval size in tone and non-tone language speech. As in music, intervals smaller than 200 cents occur less often in tone language cultures (48.7% vs. 60.3%; *t* = −5.6, *P*<0.001), whereas larger intervals occur more often (51.3% vs. 39.7%; *t* = 5.6, *P*<0.001). (See Figure S6 for breakdown by individual language).

**Table 1:** Melodic Intervals Size Statistics for the most commonly occurring intervals. (Independent – samples t-tests).

Interval size (Cents)	*n_1_*	*n_2_*	df	*t*-value	*P*- value
0	90	90	178	*t* = −6.5	*P*<0.001
100	90	90	178	*t* = −10.1	*P*<0.001
200	90	90	178	*t* = 5.3	*P*<0.001
300	90	90	178	*t* = 7.5	*P*<0.001
400	90	90	178	*t* = −7.4	*P*<0.001
500	90	90	178	*t* = 4.3	*P*<0.001

Statistics for the comparisons of the most commonly occurring melodic interval sizes in tone and non-tone language music databases; *n_1_* and *n_2_* refer to the sample sizes of tone and non-tone language music databases. (All comparisons were made with the two-tailed independent samples t-test, α-level adjusted using the Bonferroni method).

## Discussion

The music of tone and non-tone language cultures is tonally distinct, as are the languages spoken by their members. To explore the possible relationship between music and speech across cultures, we assessed the pitch dynamics of these modes of expression in three cultural groups that use tone languages (Chinese, Thai and Vietnamese) and three that use non-tone languages (American, French and German). The patterns apparent in music parallel those in speech. Thus the music of tone language cultures changes pitch direction more frequently and employs larger melodic intervals. Similarly, the speech of tone language cultures changes pitch direction more frequently and employs larger prosodic intervals. Presumably tone language speech exhibits these characteristics because the lexical meaning of each syllable in a tone language is tonally determined [15]. Consequently adjacent syllables often have different pitch contours and levels resulting in more frequent changes in pitch direction and larger pitch changes between syllables. In contrast, given that very few syllables in non-tone languages are distinguished in this way, changes in pitch direction should be less frequent and pitch changes between syllables should be smaller. 

The only exception to this pattern is the use of major thirds. Despite the greater frequency of larger prosodic intervals (≥200 cents) in the speech of tone compared to non-tone languages, there are fewer melodic major thirds (400 cents) in the music of tone language cultures (see Figure 2). A possible reason for this anomaly is scale usage. The traditional music of the tone language speaking cultures examined tends to use pentatonic scales [20]–[22], whereas traditional music of non-tone language speaking cultures examined tends to use heptatonic scales [2], [23]. Any particular scale affords different opportunities for particular melodic intervals to arise. Thus, in comparison with the major heptatonic scale, the major pentatonic scale offers approximately 6% fewer opportunities for major thirds despite an 11% increase in opportunities for larger melodic intervals (200–500 cents) overall (see Figure S7). This difference in opportunity for larger intervals may also be related to the larger number of melodic slope reversals in tone language music, since large melodic intervals tend to be followed by changes in the direction of pitch contour [16]. However, the use of different scales (and its implications for melodic structure) in different cultures raises the further question of why a given culture might favor a particular scale. Scale preferences appear to be based in part on the similarity of a set of tones to a harmonic series [6]. Consequently, the preference for pentatonic major scales in the music of the tone language speaking cultures examined could reflect the desire for a harmonically coherent set of notes that also uses relatively large melodic intervals to endorse speech similarity more specifically (see Text S1).

In sum, co-variation of tonal characteristics in the music and speech of the tone and non-tone language speaking cultures we examined indicates an intimate relationship between these two modes of social communication, providing a way of explaining at least some aesthetic preferences in biological terms.

## Materials and Methods

### Music Databases

Monophonic folk melodies from tone and non-tone language speaking cultures, all of which could be either played on an instrument or sung, were obtained from scores and MIDI format files obtained principally from National University of Singapore and the Singapore National Library Board. The tone language database comprised 50 traditional Mandarin, 20 traditional Thai, and 20 traditional Vietnamese melodies. The non-tone language database comprised 50 traditional American, 20 traditional French, and 20 traditional German melodies. To mitigate cross-cultural contamination by modern media, all compositions pre-dated 1900, often by hundreds of years.

### Speech Databases

Tone and non-tone speech samples were acquired by recording monologues read by native speakers of each of the 6 relevant languages (i.e. Standard Mandarin, Thai, and Vietnamese; American English, French, and German). Each speaker read 5 emotionally neutral monologues translated into the appropriate language (Figure S1). Prior to recording, all participants practiced reading the monologues out loud under supervision, the only instruction being to speak as if in normal conversation. The tone language database comprised recordings of 20 Standard Mandarin speakers (10 females), 10 Thai speakers (6 females), and 10 Vietnamese speakers (6 females); the non-tone language database comprised 20 American English speakers (10 females), 10 French speakers (5 females), and 10 German speakers (4 females).

### Data Analysis

The analysis of music focused on melodic slope reversals, and melodic interval size. Melodic slope reversals were defined as any change in the direction of the pitch contour of a melody. For each melody, the number of local minima and maxima was tabulated and divided by the total number of notes; this value was multiplied by 100 to give the incidence of melodic slope reversals per 100 notes. Melodic interval size was defined as the pitch difference (in cents) between adjacent notes (see Text S1 and Figure S2 for details). For each music database the distribution of interval sizes was determined separately for each melody and then averaged. These results are reported in terms of absolute interval size because the distributions of interval sizes for descending and ascending intervals were broadly similar in both musical databases (Figure S4A). 

The analysis of speech focused on two aspects of pitch dynamics analogous to those examined in music: prosodic slope reversals, and prosodic interval size. Prosodic slope reversals were defined as any change in the direction of the simplified pitch contour of a speech recording (as given by the Prosogram algorithm; see Text S1). For each speaker, the number of local minima and maxima was tabulated and divided by the total number of syllables; this value was multiplied by 100 to give the incidence of prosodic slope reversals per 100 syllables. Prosodic interval size was defined as the pitch difference (in cents) between the final and beginning pitch levels of adjacent syllables (see Text S1 and Figure S3). For each speech database, the distribution of intervals sizes was determined separately for each speaker and then averaged. As with music, the results of this analysis are reported in terms of absolute interval size because the distributions interval sizes for descending and ascending interval were broadly similar in both speech databases (Figure S4B).

## Supporting Information

Figure S1 **An example English monologue from the speech database with translations in the languages examined.**
(TIF)

Figure S2 **Music analysis.** (A) Musical notation of the traditional American melody “Home on the range”. (B) The same melody reformatted for analysis. Notes are represented by black bars with open circles marking their beginnings and endings. The length of each bar is proportional to the duration of the note. Local maxima (max) and minima (min) indicate slope reversals in the melodic pitch contour; melodic interval size (i) is the vertical distance between successive notes. Dashed lines have been added to aid visualization of the melodic pitch contour.
(TIF)

Figure S3 **Speech analysis.** (A) Recording of the sentence “I applied for a job that would give me a good work experience” spoken in American English (B) Fundamental frequency (F0) of the recording in (A) over time (segments lacking F0 values are not periodic). (C) F0s in Panel (B) segmented into syllables with simplified contours. Syllables are represented by black bars with open circles marking their beginnings and endings. (D) Output of the Prosogram analysis. Local maxima (max) and minima (min) indicate slope reversals in the prosodic pitch contour; prosodic interval size (i) is the vertical distance between successive syllables. Dashed lines have been added to aid visualization of the prosodic pitch contour.
(TIF)

Figure S4 **Interval size in the music and speech of tone and non-tone language speaking cultures sorted into descending and ascending.** (A) The distributions of descending (left panel) and ascending (right panel) melodic interval sizes per melody in the tone (red) and non-tone (blue) language music databases. Unisons (0 cents) are shown in both panels. (B) The distributions of descending (left panel) and ascending (right panel) prosodic interval sizes per speaker in the tone (red) and non-tone (blue) language speech databases. The left panel inset shows the percentages of large (≥200 cents) vs. small (<200 cents) descending prosodic intervals (the vertical dashed line in the left panel separates these groups). The right panel inset shows the percentages of small (<200 cents) vs. large (≥200 cents) ascending prosodic intervals (the vertical dashed line in the right panel separates these groups. Error bars indicate +/−2x standard errors to indicate 95% confidence intervals. (**  =  *P*<0.001; all comparisons were made using two-tailed independent samples *t*-test, α-level adjusted using the Bonferroni method).
(TIF)

Figure S5 **Slope reversals in the music and speech of tone and non-tone language speaking cultures sorted by the individual languages examined.** (A) Box plot showing the distribution of the number of melodic slope reversals per melody (normalized as the number of reversals per 100 notes per melody) in the Mandarin, Thai, and Vietnamese melodies (red), and in the English, French, and German melodies (blue). Horizontal yellow lines indicate medians; colored boxes specify inter-quartile ranges and dashed lines the ranges without outliers. (B) Box plot of the number of prosodic slope reversals per speaker (normalized as the number of reversals per 100 syllables per speaker) in the Mandarin, Thai, and Vietnamese speech (red) and English, French, and German speech (blue). Format is the same as in (A). Crosses indicate outliers (defined as greater/lesser than 1.5x the inter-quartile range). See Table S1 for statistics. (All comparisons were made with the Mann-Whitney U-test, α = 0.05, two-tailed.)
(TIF)

Figure S6 **Interval size in the music and speech of tone and non-tone language speaking cultures sorted by the individual languages examined.** (A) The distribution of absolute melodic interval sizes per melody in Mandarin, Thai, and Vietnamese melodies (red), and in the English, French, and German melodies (blue). (B) The distributions of absolute prosodic interval sizes per speaker in the Mandarin, Thai, and Vietnamese speakers (red), and the English, French, and German speakers (blue). See Table S2 for statistics.
(TIF)

Figure S7 **Comparison of melodic intervals arising from pentatonic vs. heptatonic scale structure.** (A) Illustration of heptatonic and pentatonic scales on piano keyboards (circles indicate scale notes). The pattern within a single octave repeats in each octave. (B) Histogram of the percentages of all possible intervals ≤ 500 cents arising from analysis of the scales in (A). Red bars represent pentatonic scale percentages, and blue bars heptatonic scale percentages.
(TIF)

Table S1 **Statistics comparing the number of slope reversals in tone and non-tone language music and speech databases for each possible pair of the cultures examined.** (A) Statistics for melodic slope reversals in music. (B) Statistics for prosodic slope reversals in speech; *n*_1_ and *n*_2_ refer to the sample sizes of group 1 and group 2. (All comparisons were made using the Mann-Whitney U-test, α = 0.05, two-tailed)
(DOC)

Table S2 **Statistics for the comparisons of interval size distributions in the tone and non-tone language music and speech databases for each possible pair of the cultures examined.** (A) Statistics for melodic interval size distributions. (B) Statistics for prosodic interval size distributions; *n*_1_ and *n*_2_ refer to the sample sizes of groups 1 and 2. (All comparisons were made with the independent samples t-test, α = 0.05, two-tailed.)
(DOC)

Text S1 **Supporting Methods and Results.** This file gives an overview of the methods and results used in this manuscript.
(DOC)

## References

[1] Von Helmholtz HLF (1954). On the sensations of tone..

[2] Dean B (1985). That ‘howling’ music: Japanese hougaku in contrast to Western art music.. Monumenta Nipponica.

[3] Haviland WA, Prins HEL, Walrath D, McBride B (2007). Cultural anthropology: the human challenge..

[4] Juslin PN, Laukka P (2003). Communication of emotions in vocal expression and music performance: different channels, same code?. Psychological Bulletin.

[5] Schwartz D, Howe CQ, Purves D (2003). The statistical structure of human speech sounds predicts musical universals.. Journal of Neuroscience.

[6] Gill KZ, Purves DA (2009). Biological rationale for musical scales.. PLoS ONE.

[7] Bowling D, Gill K, Choi J, Prinz J, Purves D (2010). Major and minor music compared to excited and subdued speech.. Journal of the Acoustical Society of America.

[8] Patel AD, Iversen JR, Rosenberg JC (2006). Comparing the rhythm and melody of speech and music: the case of British English and French.. Journal of the Acoustical Society of America.

[9] McGowan RW, Levitt AG (2011). A comparison of Rhythm in English dialects and music.. Music Perception.

[10] Crystal D (1997). The Cambridge Encyclopedia of Language, 2^nd^ Edition..

[11] Abramson AS (1962). The vowels and tones of standard Thai: acoustical measurements and experiments.. International Journal of American Linguistics.

[12] Hirst D, Di Cristo A (1999). Intonation Systems: A Survey of Twenty Languages..

[13] Brown K, Ogilive S (2008). Concise encyclopedia of languages of the world..

[14] Brazil D (1997). The Communicative Value of Intonation in English..

[15] Eady SJ (1982). Differences in the F0 patterns of speech: tone language versus stress language.. Language and Speech.

[16] Von Hippel P, Huron D (2000). Why do skips precede reversals? The effect of Tessitura on melodic structure.. Music Perception.

[17] Dowling WJ, Harwood DL (1986). Music cognition..

[18] Vos P, Troost J (1989). Ascending and Descending Melodic Intervals: Statistical Findings and Their Perceptual Relevance.. Music Perception.

[19] Thompson WF, Graham P, Russo FA (2005). Seeing music performance: Visual influences on perception and experience.. Semiotica,.

[20] Morton D (1976). The Traditional Music of Thailand..

[21] Ho LT, Han KH (1982). On Chinese scales and national modes.. Asian music.

[22] Keefe DH, Burns EM, Nguyen P (1991). Vietnamese modal scales of the Dan Tranh.. Music perception.

[23] Barry P (1909). Folk music in America.. Journal of American folk-lore.

