# Supplementary figures and images for: Co-Variation of Tonality in the Music and Speech of Different Cultures
Source: PLoS One. 2011 May 27;6(5):e20160. doi: 10.1371/journal.pone.0020160 (PMC3103533; doi:10.1371/journal.pone.0020160)

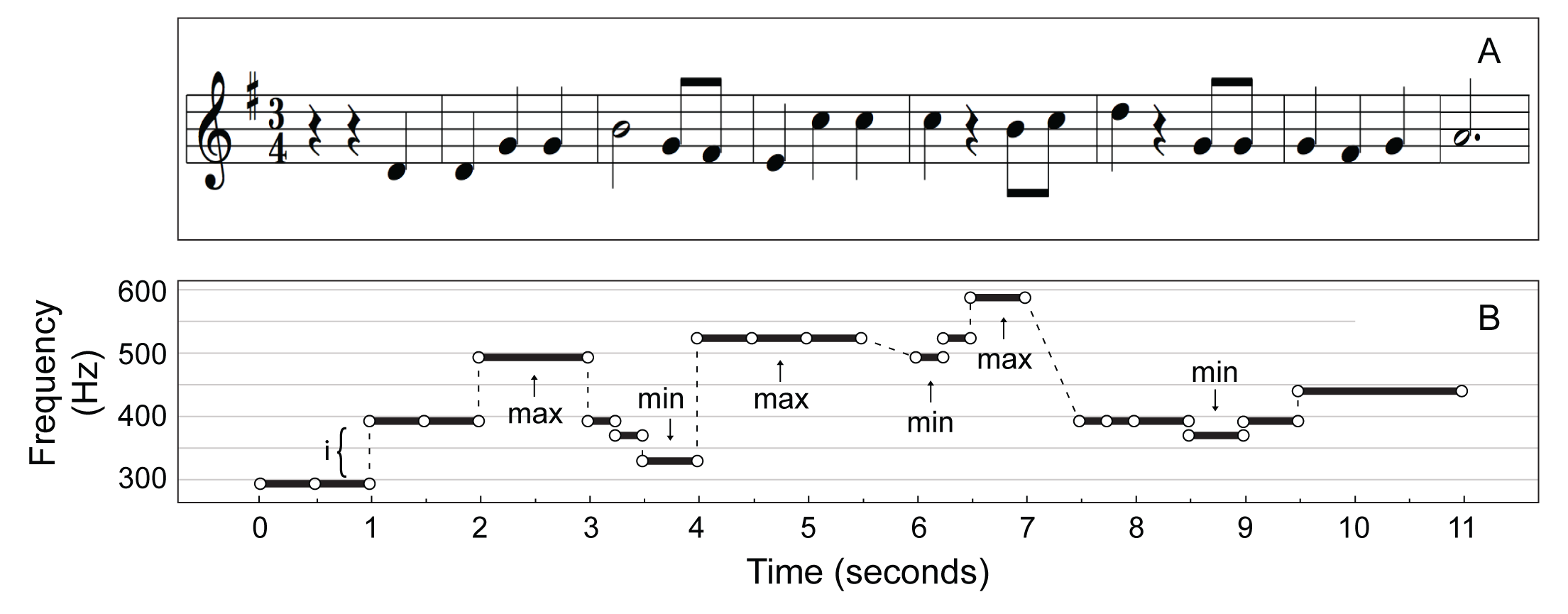

Supplement: Figure S1 — An example English monologue from the speech database with translations in the languages examined. (TIF) [file pone.0020160.s001.tif]

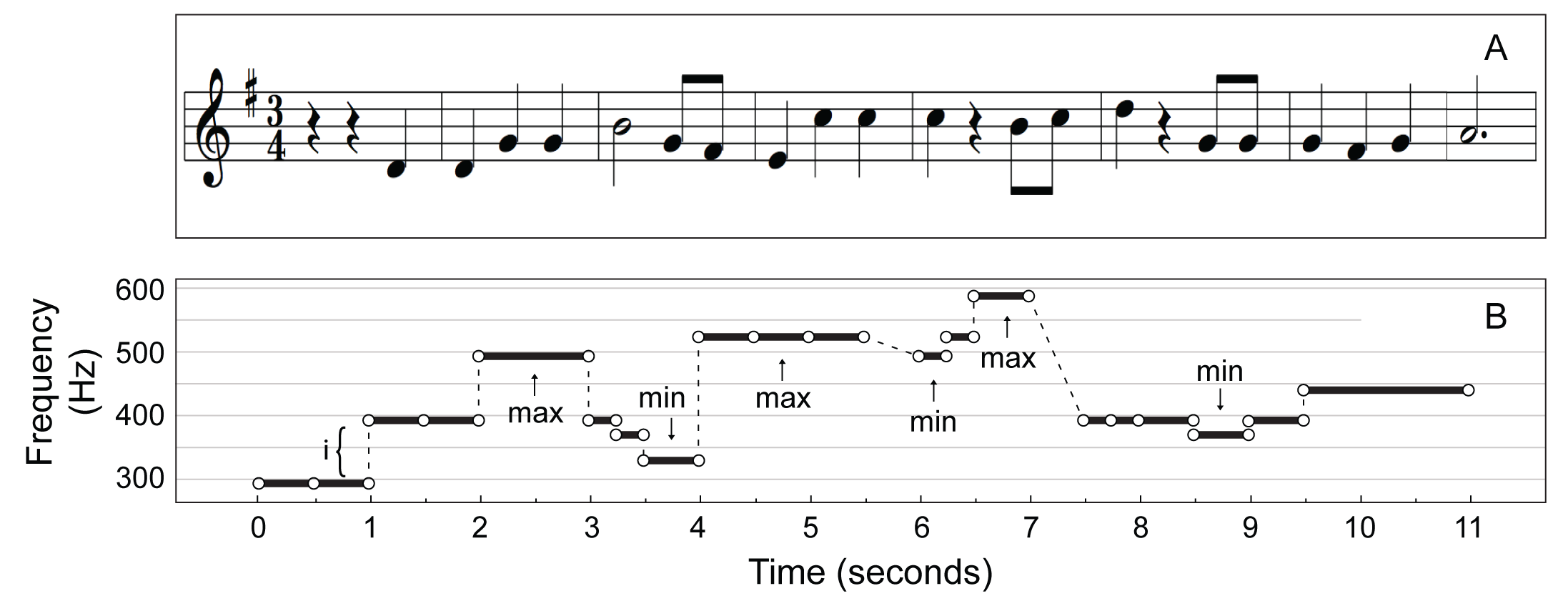

Supplement: Figure S2 — Music analysis. (A) Musical notation of the traditional American melody “Home on the range”. (B) The same melody reformatted for analysis. Notes are represented by black bars with open circles marking their beginnings and endings. The length of each bar is proportional to the duration of the note. Local maxima (max) and minima (min) indicate slope reversals in the melodic pitch contour; melodic interval size (i) is the vertical distance between successive notes. Dashed lines have been added to aid visualization of the melodic pitch contour. (TIF) [file pone.0020160.s002.tif]

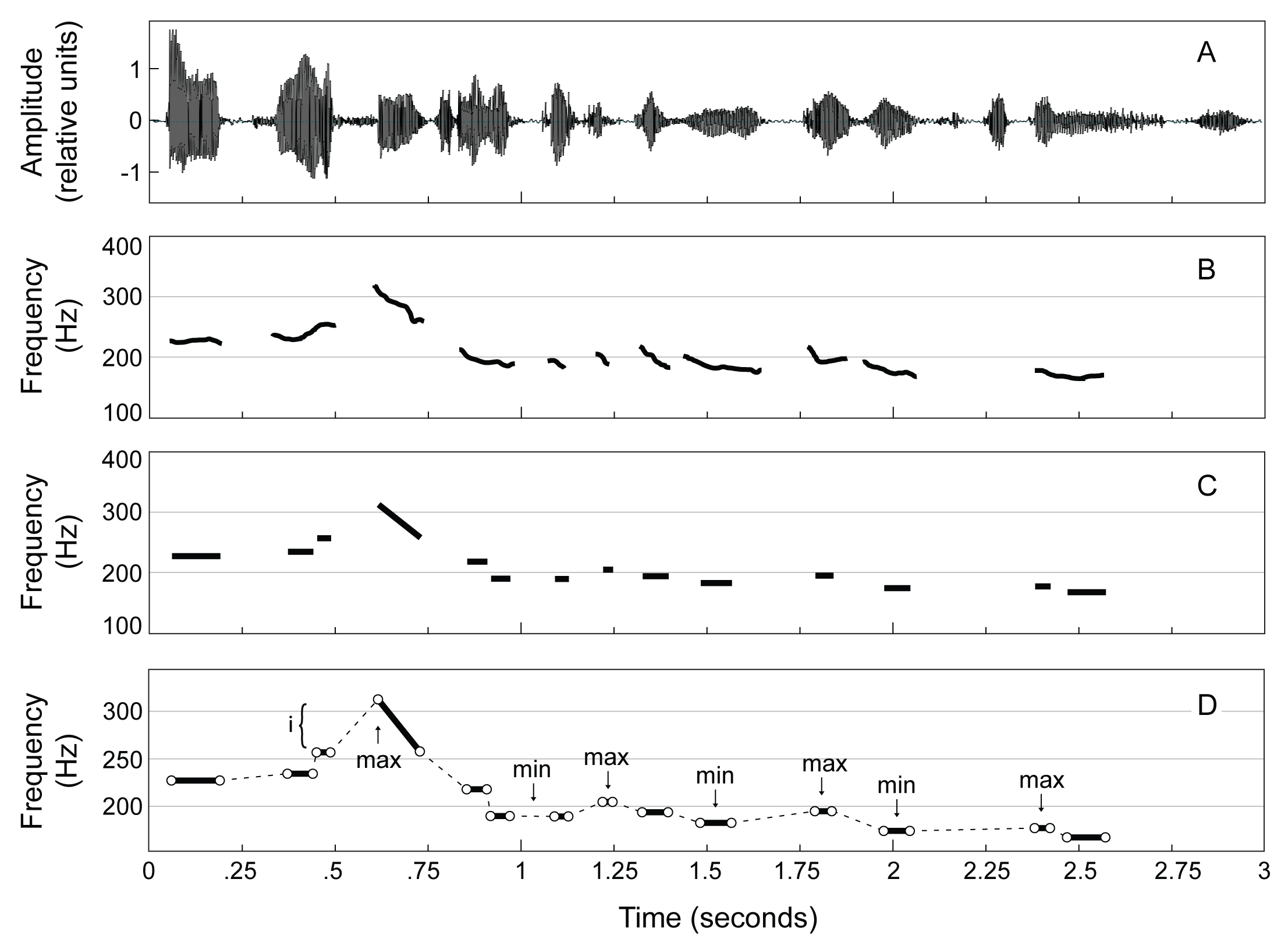

Supplement: Figure S3 — Speech analysis. (A) Recording of the sentence “I applied for a job that would give me a good work experience” spoken in American English (B) Fundamental frequency (F0) of the recording in (A) over time (segments lacking F0 values are not periodic). (C) F0s in Panel (B) segmented into syllables with simplified contours. Syllables are represented by black bars with open circles marking their beginnings and endings. (D) Output of the Prosogram analysis. Local maxima (max) and minima (min) indicate slope reversals in the prosodic pitch contour; prosodic interval size (i) is the vertical distance between successive syllables. Dashed lines have been added to aid visualization of the prosodic pitch contour. (TIF) [file pone.0020160.s003.tif]

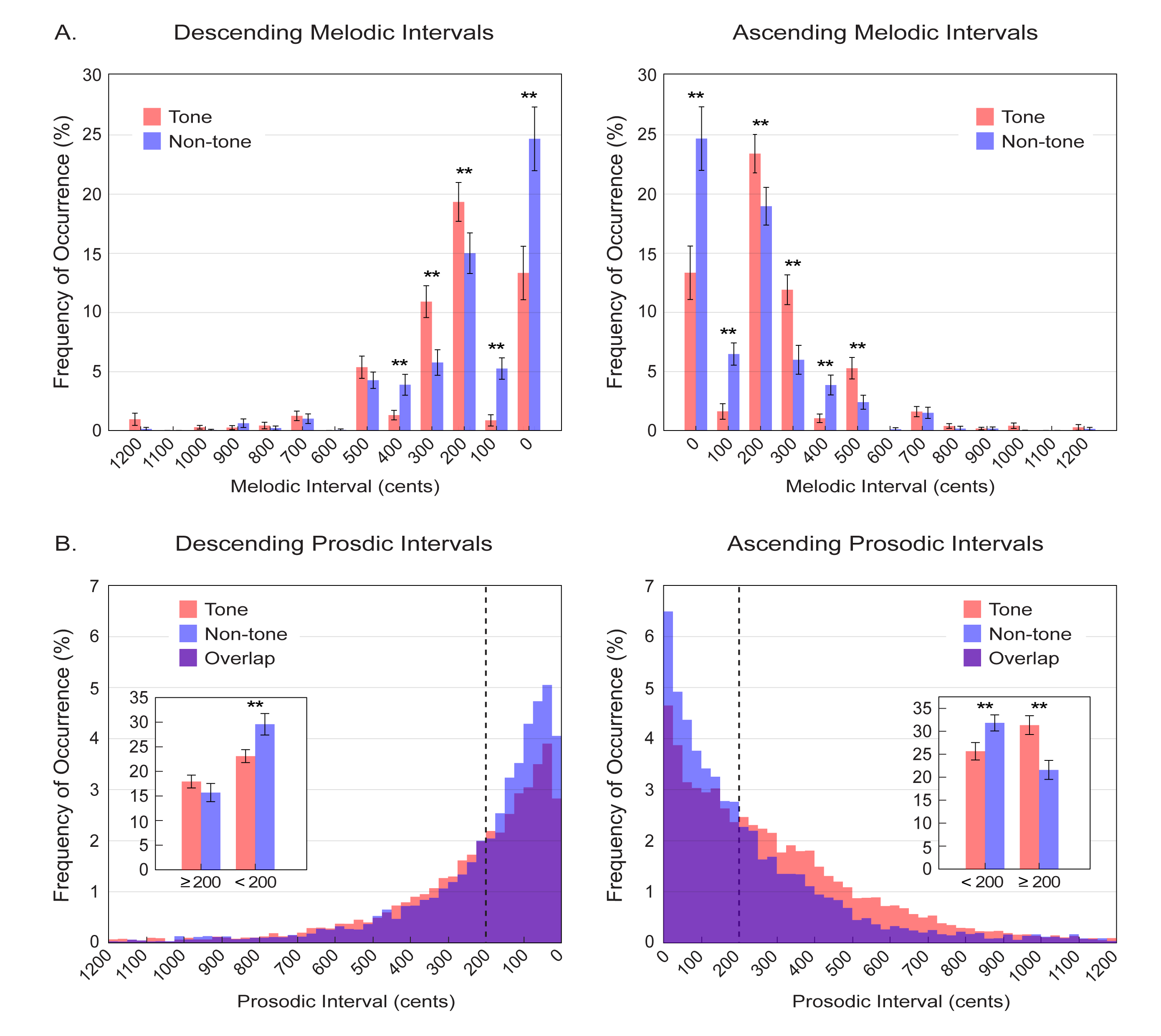

Supplement: Figure S4 — Interval size in the music and speech of tone and non-tone language speaking cultures sorted into descending and ascending. (A) The distributions of descending (left panel) and ascending (right panel) melodic interval sizes per melody in the tone (red) and non-tone (blue) language music databases. Unisons (0 cents) are shown in both panels. (B) The distributions of descending (left panel) and ascending (right panel) prosodic interval sizes per speaker in the tone (red) and non-tone (blue) language speech databases. The left panel inset shows the percentages of large (≥200 cents) vs. small (<200 cents) descending prosodic intervals (the vertical dashed line in the left panel separates these groups). The right panel inset shows the percentages of small (<200 cents) vs. large (≥200 cents) ascending prosodic intervals (the vertical dashed line in the right panel separates these groups. Error bars indicate +/−2x standard errors to indicate 95% confidence intervals. (** = P<0.001; all comparisons were made using two-tailed independent samples t-test, α-level adjusted using the Bonferroni method). (TIF) [file pone.0020160.s004.tif]

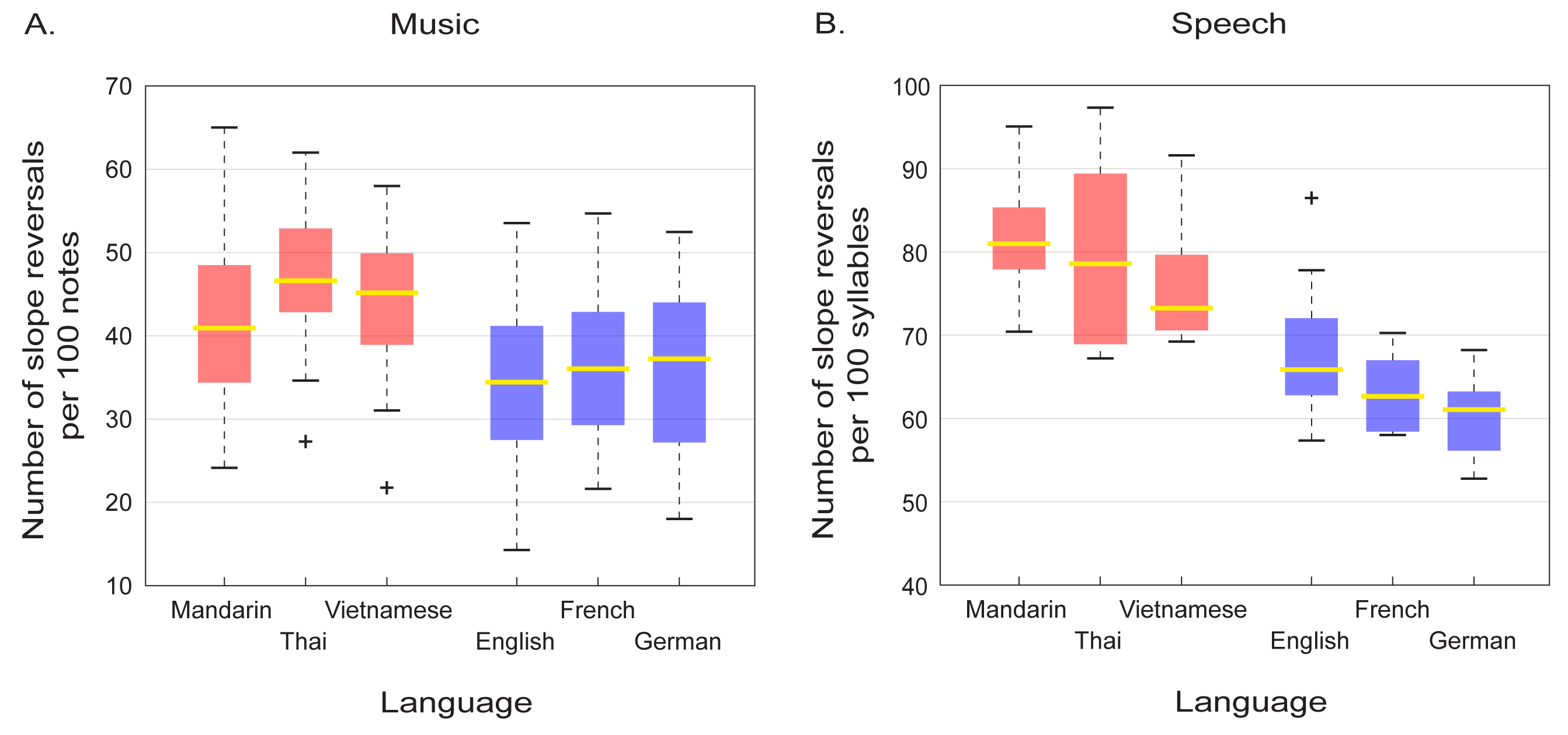

Supplement: Figure S5 — Slope reversals in the music and speech of tone and non-tone language speaking cultures sorted by the individual languages examined. (A) Box plot showing the distribution of the number of melodic slope reversals per melody (normalized as the number of reversals per 100 notes per melody) in the Mandarin, Thai, and Vietnamese melodies (red), and in the English, French, and German melodies (blue). Horizontal yellow lines indicate medians; colored boxes specify inter-quartile ranges and dashed lines the ranges without outliers. (B) Box plot of the number of prosodic slope reversals per speaker (normalized as the number of reversals per 100 syllables per speaker) in the Mandarin, Thai, and Vietnamese speech (red) and English, French, and German speech (blue). Format is the same as in (A). Crosses indicate outliers (defined as greater/lesser than 1.5x the inter-quartile range). See Table S1 for statistics. (All comparisons were made with the Mann-Whitney U-test, α = 0.05, two-tailed.) (TIF) [file pone.0020160.s005.tif]

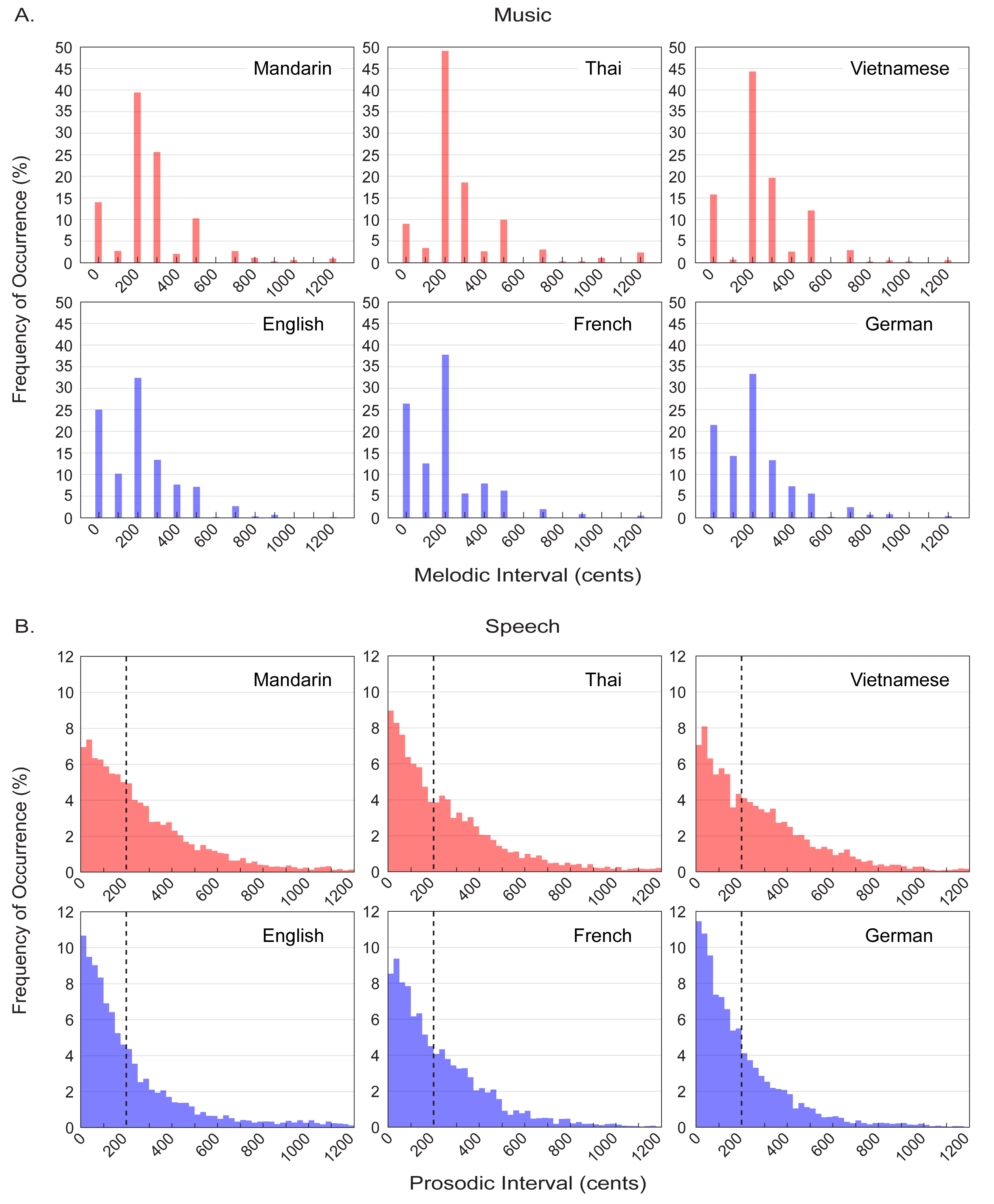

Supplement: Figure S6 — Interval size in the music and speech of tone and non-tone language speaking cultures sorted by the individual languages examined. (A) The distribution of absolute melodic interval sizes per melody in Mandarin, Thai, and Vietnamese melodies (red), and in the English, French, and German melodies (blue). (B) The distributions of absolute prosodic interval sizes per speaker in the Mandarin, Thai, and Vietnamese speakers (red), and the English, French, and German speakers (blue). See Table S2 for statistics. (TIF) [file pone.0020160.s006.tif]

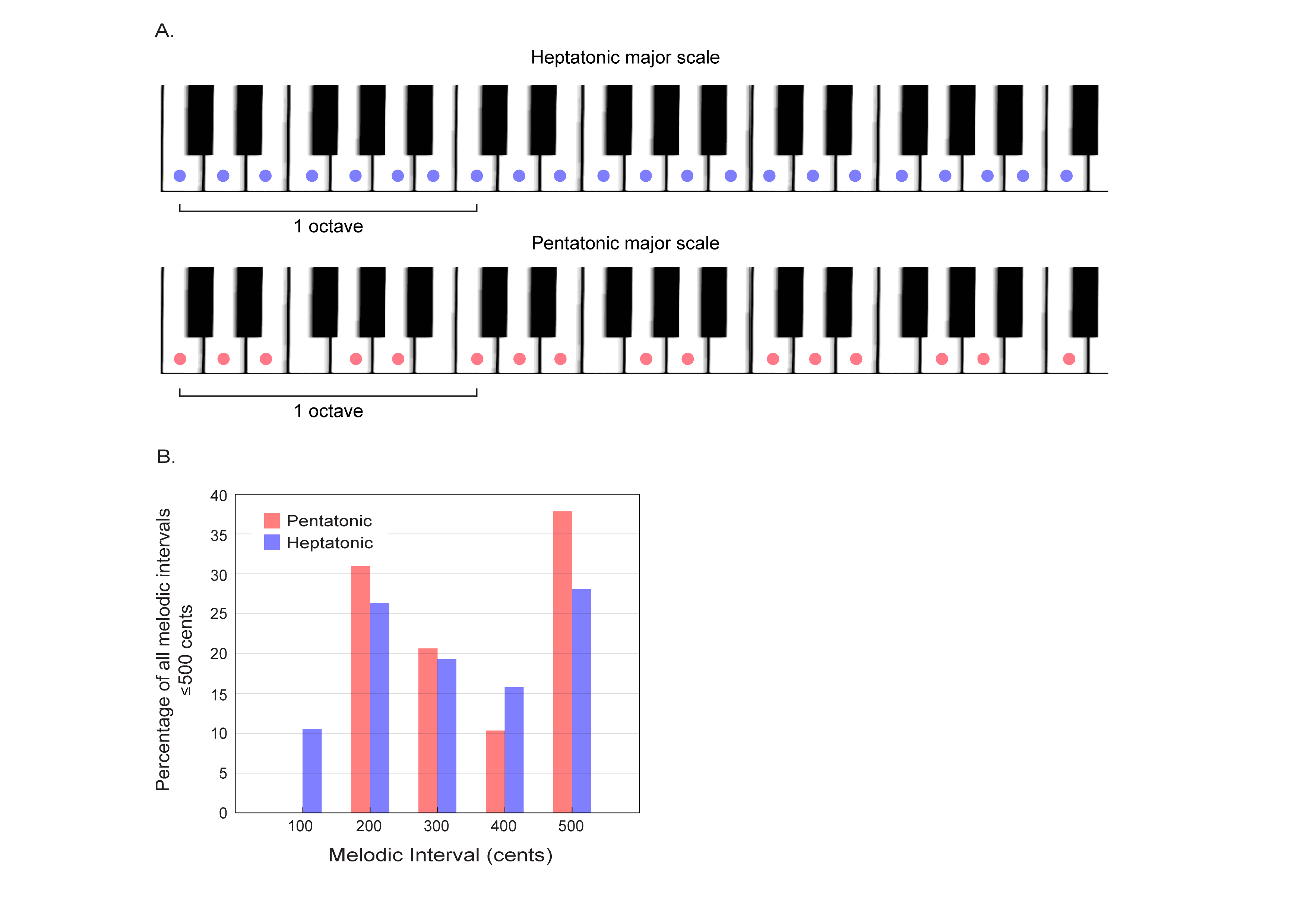

Supplement: Figure S7 — Comparison of melodic intervals arising from pentatonic vs. heptatonic scale structure. (A) Illustration of heptatonic and pentatonic scales on piano keyboards (circles indicate scale notes). The pattern within a single octave repeats in each octave. (B) Histogram of the percentages of all possible intervals ≤ 500 cents arising from analysis of the scales in (A). Red bars represent pentatonic scale percentages, and blue bars heptatonic scale percentages. (TIF) [file pone.0020160.s007.tif]
