# Supplementary material for: Co-Variation of Tonality in the Music and Speech of Different Cultures
Source: PLoS One. 2011 May 27;6(5):e20160. doi: 10.1371/journal.pone.0020160 (PMC3103533; doi:10.1371/journal.pone.0020160)
Supplement: Table S1 — Statistics comparing the number of slope reversals in tone and non-tone language music and speech databases for each possible pair of the cultures examined. (A) Statistics for melodic slope reversals in music. (B) Statistics for prosodic slope reversals in speech; n 1 and n 2 refer to the sample sizes of group 1 and group 2. (All comparisons were made using the Mann-Whitney U-test, α = 0.05, two-tailed) (DOC) [file pone.0020160.s008.doc]

**Table S1. (A) Melodic Slope Reversal Statistics (Mann-Whitney U tests)**

| **Melodies (Group1)** | **Melodies (Group2)** | ***n_1_*** | ***n_2_*** | ***U* -value** | ***P*- value** |
| --- | --- | --- | --- | --- | --- |
| Mandarin | English | 50 | 50 | 2952 | *p<*0.01 |
| Mandarin | French | 50 | 20 | 556.5 | *p<*0.05 |
| Mandarin | German | 50 | 20 | 556.5 | *p<*0.05 |
| Thai | English | 20 | 50 | 1015.5 | *p<*0.001 |
| Thai | French | 20 | 20 | 520 | *p<*0.01 |
| Thai | German | 20 | 20 | 526.5 | *p<*0.01 |
| Vietnamese | English | 20 | 50 | 956 | *p<*0.01 |
| Vietnamese | French | 20 | 20 | 502.5 | *p<*0.05 |
| Vietnamese | German | 20 | 20 | 504 | *p<*0.05 |

Table S1. (B) Prosodic Slope Reversal Statistics (Mann-Whitney U tests)

| **Speakers (Group1)** | **Speakers (Group2)** | ***n_1_*** | ***n_2_*** | ***U*-value** | ***P*- value** |
| --- | --- | --- | --- | --- | --- |
| Mandarin | English | 20 | 20 | 580 | p<0.001 |
| Mandarin | French | 20 | 10 | 55 | p<0.001 |
| Mandarin | German | 20 | 10 | 55 | p<0.001 |
| Thai | English | 10 | 20 | 227 | p<0.01 |
| Thai | French | 10 | 10 | 151 | p<0.001 |
| Thai | German | 10 | 10 | 154 | p<0.001 |
| Vietnamese | English | 10 | 20 | 217 | p<0.01 |
| Vietnamese | French | 10 | 10 | 153 | p<0.001 |
| Vietnamese | German | 10 | 10 | 155 | p<0.001 |
