# Supplementary material for: Co-Variation of Tonality in the Music and Speech of Different Cultures
Source: PLoS One. 2011 May 27;6(5):e20160. doi: 10.1371/journal.pone.0020160 (PMC3103533; doi:10.1371/journal.pone.0020160)
Supplement: Table S2 — Statistics for the comparisons of interval size distributions in the tone and non-tone language music and speech databases for each possible pair of the cultures examined. (A) Statistics for melodic interval size distributions. (B) Statistics for prosodic interval size distributions; n 1 and n 2 refer to the sample sizes of groups 1 and 2. (All comparisons were made with the independent samples t-test, α = 0.05, two-tailed.) (DOC) [file pone.0020160.s009.doc]

**Table S2. (A) Melodic Intervals Size** Statistics (Independent – samples t-tests)

| **Melodies (Group1)** | **Melodies (Group2)** | ***n_1_*** | ***n_2_*** | **df** | **t-value** | ***P*- value Intervals < 200 cents** | ***P*- value Intervals ≥ 200 cents** |
| --- | --- | --- | --- | --- | --- | --- | --- |
| Mandarin | English | 50 | 50 | 98 | t = 8.1 | *P*<0.001 | *P* <0.001 |
| Mandarin | French | 50 | 20 | 68 | t = 7.6 | *P* <0.001 | *P* <0.001 |
| Mandarin | German | 50 | 20 | 68 | t = 6.3 | *P* <0.001 | *P* <0.001 |
| Thai | English | 20 | 50 | 68 | t = 7.9 | *P* <0.001 | *P* <0.001 |
| Thai | French | 20 | 20 | 38 | t = 8.6 | *P* <0.001 | *P* <0.001 |
| Thai | German | 20 | 20 | 38 | t = 7.0 | *P* <0.001 | *P* <0.001 |
| Vietnamese | English | 20 | 50 | 68 | t = 5.1 | *P* <0.001 | *P* <0.001 |
| Vietnamese | French | 20 | 20 | 38 | t = 4.8 | *P* <0.001 | *P* <0.001 |
| Vietnamese | German | 20 | 20 | 38 | t = 3.9 | *P* <0.001 | *P* <0.001 |

**Table S2. (B) Prosodic Intervals Size** Statistics (Independent – samples t-tests)

| **Speakers (Group1)** | **Speakers (Group2)** | ***n_1_*** | ***n_2_*** | **df** | **t-value** | ***P*- value Intervals < 200 cents** | ***P*- value Intervals ≥ 200 cents** |
| --- | --- | --- | --- | --- | --- | --- | --- |
| Mandarin | English | 20 | 20 | 38 | t = 4.4 | *P*<0.001 | *P*<0.001 |
| Mandarin | French | 20 | 10 | 28 | t = 1.8 | *P* = 0.08 | *P* = 0.08 |
| Mandarin | German | 20 | 10 | 28 | t = 4.0 | *P*<0.001 | *P*<0.001 |
| Thai | English | 10 | 20 | 28 | t = 2.9 | *P*<0.01 | *P*<0.01 |
| Thai | French | 10 | 10 | 18 | t = 1.0 | *P*= 0.35 | *P*= 0.35 |
| Thai | German | 10 | 10 | 18 | t = 2.9 | *P*<0.01 | *P*<0.01 |
| Vietnamese | English | 10 | 20 | 28 | t = 4.8 | *P*<0.001 | *P*<0.001 |
| Vietnamese | French | 10 | 10 | 18 | t = 2.2 | *P*<0.05 | *P*<0.05 |
| Vietnamese | German | 10 | 10 | 18 | t = 4.4 | *P*<0.001 | *P*<0.001 |
