# Supplementary material for: Co-Variation of Tonality in the Music and Speech of Different Cultures
Source: PLoS One. 2011 May 27;6(5):e20160. doi: 10.1371/journal.pone.0020160 (PMC3103533; doi:10.1371/journal.pone.0020160)
Supplement: Text S1 — Supporting Methods and Results. This file gives an overview of the methods and results used in this manuscript. (DOC) [file pone.0020160.s010.doc]

**Supporting Methods**

Music Data

Out of the 180 melodies in the music databases, 150 were only available as scores and needed to be converted into MIDI format (the other 30 were already in MIDI format). These melodies were played on a Yamaha PSR E213 MIDI keyboard, recorded by Finale SongWriter (version 2010) and saved in MIDI format. For all melodies the tempo was held constant at 120 beats per minute. Melodies from the tone language cultures contained 18 to 89 notes (mean= 48.8, s.d.= 15.5) and those from non-tone language cultures 19 to 74 notes (mean= 45.6, s.d.= 13.1). All melodies were tuned in equal temperament.

Speech Data

Each speaker was required to have lived in the country of their native language for at least 10 years, and to have continued speaking it on a daily basis. Examples of the monologues read by speakers are shown in Figure S1. Participants ranged in age from 19-68 years. Depending of the availability of the speakers, recordings were made either in a quiet room in the field or in an Eckel CL-12A audiometric room at Duke-NUS using an Audio-Technica AT4049a omni-directional capacitor microphone and a Marantz PMD670 solid-state recorder (Martel Electronics, Yorba Linda CA) at a sampling rate of 22.05kHz. These recordings were saved as WAV files on a Transcend flash memory card and transferred to an Apple MacBook Pro for analysis.

Analysis of Music

MIDI files were analyzed using MIDI Toolbox 1.0.1 [1] for MATLAB R2009a [2]. This allowed extraction of the difference (+/-) between adjacent notes in semitones, which was in turn used to tabulate the number of melodic slope reversals and the size of melodic intervals in cents (1 semitone= 100 cents; Figure S2).

Analysis of Speech

Speech recordings were processed using Praat (3; version 4.5) and Prosogram [4] (version 2.7; instantiated in Praat). Fundamental frequency (F0) was calculated in 10ms time-steps using Praat’s “to pitch” autocorrelation algorithm [5]. Different pitch floors and ceilings were used for males and females as recommended [3] (pitch floor for male/female = 75Hz/100Hz, pitch ceiling for male/female = 300Hz/500Hz). All other parameters were set to their default values. F0 detection errors attributed to unvoiced speech (e.g., fricatives generating a fundamental frequency) and other anomalies that occur in any speech analysis [6] were excluded by direct inspection. There were never more than ~30 such errors in the approximately 4000-4900 voiced speech data points recorded for each speaker (errors consisting of ~3-5 data points). The corrected F0 values were then used as input to Prosogram.

The Prosogram algorithm simplifies the F0 contour and makes it comparable to music. It does this by marking syllabic nuclei boundaries (based on intensity minima) in the F0 contour, applying a low pass filter to smooth out rapid F0 changes, and flattening syllable contours whose rate of F0 change is less than the ”glissando threshold” [7,8]. All Prosogram parameters were set to default values with the exception of the glissando threshold, which was set at .16 semitones/second instead of .32 semitones/second. This was done to preserve semantically relevant intra-syllabic pitch variation in tone-language speech, which was found to be obscured by the .32 semitones/second threshold.

The accuracy with which the Prosogram algorithm identified syllables was found to be ~83%. This was determined by comparing its results with those obtained by manual transcription of syllables using spectrogram-based segmentation [9] in a subset of speech recordings (one monologue per speaker for all American English and Standard Mandarin speakers). Accuracy was defined as the number of syllables correctly identified by automatic segmentation ($C$), taken as a percentage of the total number of syllables identified by automatic segmentation ($T$) plus the number of syllables missed by automatic segmentation ($M$).

Descending and ascending intervals in music and speech

Figure S4A shows the distributions of melodic interval sizes in the tone and non-tone language music databases sorted into descending and ascending intervals. The pattern is similar in both directions. In music from tone compared to non-tone language cultures, descending melodic intervals smaller than a major second (200 cents) occur significantly less (14.2% vs 29.8%, *t* =-9.03, *P*<0.001), whereas descending melodic intervals equal to or larger than a major second occur significantly more (40% vs 30.8%, *t*= 8.04, *P*<0.001). Likewise, music from tone compared to non-tone language cultures has significantly less ascending melodic intervals smaller than a major second (14.9% vs 31%, *t=* -9.20, *P*<0.001), and significantly more ascending melodic intervals equal to or larger than a major second (44.3% vs. 33%, *t*= 10.2, *P*<0.001).

Figure S4B shows the distributions of prosodic interval sizes in the tone and non-tone language speech databases sorted into descending and ascending intervals. As with the melodic intervals, the pattern for prosodic intervals is similar in both directions. In speech from tone compared to non-tone language cultures, descending prosodic intervals smaller than 200 cents occur less (23.1% vs 29.5%, *t*=-5.07, *P*<0.001), whereas descending prosodic intervals equal to or larger than 200 cents occur more (17.9% vs 15.7%, *t*=1.98, *P*= 0.051), although this difference did not reach significance at *P*= 0.05. Likewise, speech from tone compared to non-tone language cultures has significantly less ascending prosodic intervals smaller than 200 cents (25.7% vs 31.8%, *t*=-4.82, *P*<0.001), and more ascending prosodic intervals equal to or larger than 200 cents (31.3% vs 21.6%, *t*=6.71, *P*<0.001).

**Supporting Results**

Analysis of prosodic slope reversals in music and speech by individual languages

Figure S5 compares the number of slope reversals in the tone and non-tone music and speech databases sorted by the individual languages examined. The difference in the median number of melodic slope reversals is significant between melodies from each possible pair of the tone and non-tone language speaking cultures (see Table S1A). Likewise, the difference in the median number of prosodic slope reversals is significant between speakers of each possible pair of the tone and non-tone language speaking cultures (see Table S1B).

Analysis of interval size in music and speech by individual languages

Figure S6 shows the distributions of melodic and prosodic interval sizes sorted by the individual languages examined. The difference in the proportion of melodic intervals smaller than 200 cents and the difference in the proportion of melodic intervals equal to or larger than 200 cents is significant between melodies from each possible pair of the tone and non-tone language speaking cultures (see Table S2A). The difference in the proportion of prosodic intervals smaller than 200 cents and the difference in the proportion of prosodic intervals equal to or larger than 200 cents is significant between speakers of each possible pair of tone and non-tone language speaking cultures examined with the exceptions of Mandarin and French, and Thai and French (see Table S2B).

Analysis of melodic intervals in pentatonic and heptatonic scales

The contribution of scale structure to the distributions of melodic interval size observed in the tone and non-tone language music databases (see Figure 2) was examined by comparing the opportunities for different melodic intervals to arise in pentatonic versus heptatonic scales (Figure S7). Inspection of the scores in the music databases indicated that the pentatonic major scale and the heptatonic major scale describe the tonality of the majority of melodies. Approximately 64 of the 90 melodies (71%) in the tone language music database use the pentatonic major scale, and approximately 80 of the 90 melodies (89%) in the non-tone language music database use the heptatonic major scale. Other scales included the heptatonic minor scale, the pentatonic relative minor scale, and others that could not be clearly identified. Accordingly, the analysis focused on the pentatonic and heptatonic major scales. The opportunity for different melodic intervals to occur within these scales was analyzed over three octaves (as opposed to one) to reflect the octave range observed in the melodies.

The analysis for each scale was completed in three steps. First, every possible pair of notes within 3 octaves was determined. Pairings of notes with themselves were excluded, leaving 120 pairs in the pentatonic major scale and 231 pairs in the heptatonic major scale. Second, the absolute interval between each pair of notes was determined (in cents). Because of their rare occurrence in melodies (see text), intervals greater than 500 cents were excluded, leaving 29 intervals for the pentatonic major scale and 57 intervals for the heptatonic major scale. Third, the frequency of occurrence of each interval in this subset was calculated as a percentage.

This comparison shows that the pentatonic major scale affords 5.5% less opportunities for melodic major thirds to arise than the heptatonic major scale (10.3% vs. 15.8%). The comparison also shows that overall, large melodic intervals (200-500 cents) occur 10.5% more frequently in the pentatonic major scale (100% vs. 89.5%), and that small melodic intervals (less than 200 cents) occur 10.5% more frequently in the heptatonic major scale (10.5% vs. 0%). These overall differences arise from the fact that the heptatonic major scale contains semitones (100 cents) whereas the pentatonic major scale does not.

**Supporting References**

1. Eerola T, Toivianen P (2004) MIDI Toolbox: MATLAB Tools for Music Research. University of Jyväskylä: Kopijyvä, Jyväskylä, Finland. Available: <http://www.jyu.fi/hum/laitokset/musiikki/en/research/coe/materials/miditoolbox/>
2. Mathworks Inc. MATLAB (Version 2009a) (2009) The MathWorks Inc., Natick, MA.
3. Boersma P, Weenik D (2008) Praat: Doing phonetics by computer (Version 5.043). Manual and software available: <http://www.fon.hum.uva.nl/praat/>. Accessed 20^th^ October, 2010.
4. Mertens P (2004) The Prosogram: Semi-Automatic Transcription of Prosoody Based on Tonal Perception Model. Proceedings of Speech Prosody 2004, Nara (Japan).
5. Boersma P(1993) Accurate short-term analysis of the fundamental frequency and the harmonics-to-noise ratio of a sampled sound. Proceedings of the Institute of Phonetic Sciences 17: 91-110.
6. Boril H, Pollák P (2004) Direct time domain fundamental frequency estimation of speech in noisy conditions. Proceedings of European Signal Processing Conference in Vienna, Austria. September 6-10.
7. d’ Alessandro C, d' Mertens P (1995) Automatic pitch contour stylization using a model of tonal perception. Computer Speech and Language 9: 257-288.
8. d’Alessandro C, Rosset S, Rossi J (1998) The pitch of short-duration fundamental frequency glissandos. Journal of the Acoustical Society of America 104: 2339-2348.
9. Peterson GE, Lehiste I (1960) Duration of syllabic nuclei in English. Journal of the Acoustical Society of America 32:693-703.
